# Advances in PD-1/PD-L1 pathway inhibitors in the treatment of thyroid cancer: mechanisms and clinical therapeutic perspectives

**DOI:** 10.3389/fimmu.2025.1643421

**Published:** 2025-08-08

**Authors:** Xizi Li, Siyuan Gao, Chuchu Shan, Qiling Zhang, Ying Tan, Xu Yu, Jiangyi Yu

**Affiliations:** ^1^ Affiliated Hospital of Nanjing University of Chinese Medicine, Jiangsu, Nanjing, China; ^2^ Nanjing University of Chinese Medicine, Jiangsu, Nanjing, China; ^3^ Guanghua Hospital Affiliated to Shanghai University of Traditional Chinese Medicine, Shanghai, China; ^4^ Department of Endocrinology, Jiangsu Province Hospital of Chinese Medicine, Affiliated Hospital of Nanjing University of Chinese Medicine, Nanjing, China

**Keywords:** thyroid cancer, PD-1/PD-L1 inhibitors, targeted therapy, immunotherapy, review

## Abstract

Cancer of the thyroid is a endocrine cancer. Although most patients achieve favorable outcomes with surgical resection, radioactive iodine (RAI) ablation, and thyroid-stimulating hormone (TSH) suppression therapy, a subset progresses to advanced or refractory disease. Immune checkpoint inhibitors (ICIs) blocking the PD-1/PD-L1 pathway reactivate T cells, enabling them to identify and eradicate malignant cells, thus reinstating immune surveillance against tumors. This review examines PD-L1 (Programmed Death-Ligand 1) expression in thyroid cancer, exploring its underlying regulatory mechanisms. It also discusses recent advances in PD-1/PD-L1 immune checkpoint inhibition (ICI) therapy. Furthermore, the review highlights regulatory pathways modulating PD-1/PD-L1 expression, including the mTOR pathway, androgen receptor (AR), and the CKS1B/STAT3 pathway. Notably, it summarizes recent clinical developments, such as combination regimens pairing PD-L1 blockade with mutation-targeted therapies, for which the median OS of the targeted combination therapy group was 14.7 months. This therapy has achieved the longest median OS for anaplastic thyroid carcinoma (ATC) patients so far. Additionally, the review examines innovative treatment modalities, offering a thorough synthesis of the existing state and emerging trends in PD-1/PD-L1 immunotherapies.

## 1 Introduction

Thyroid cancer is a category of endocrine tumors primarily originating from follicular epithelial cells. Based on the degree of differentiation, it can be classified into: differentiated types (including papillary and follicular carcinomas), poorly differentiated type, and anaplastic type (1). The latest epidemiological investigation pointed out that thyroid carcer as one of common solid-organ malignancies, exhibiting a disproportionately elevated incidence rate in females compared to males. This sex disparity is believed to be associated with increased use of imaging diagnostics and fine-needle aspiration biopsy, as well as with estrogen receptor signaling abnormalities and reproductive factors (2–4). Although most thyroid cancers—especially differentiated subtypes—are effectively managed through surgery, RAI ablation, and TSH suppression therapy, a portion of cases progress to advanced stages or become refractory, including poorly differentiated, undifferentiated, or iodine-refractory differentiated thyroid cancers. These subtypes are resistant to conventional therapies and exhibit markedly reduced survival. Thus, the development of novel treatment strategies remains an urgent priority.

Recent advances in understanding tumor immune escape mechanisms have offered new therapeutic avenues for thyroid cancer. Immune surveillance evasion by tumor cells occurs via engagement of inhibitory checkpoint pathways, which compromise the host’s capacity to detect and clear cancerous cells. This process is a key contributor to tumor advancement and dissemination. One of the immune checkpoints is programmed cell death protein 1 (PD-1, CD279), which is encoded by PDCD1. PD-1 (CD279) is classified as a type I transmembrane glycoprotein. This immunosuppressive receptor’s architecture includes an IgV-type ectodomain, transmembrane compartment, and cytosolic elements containing ITIM and ITSM motifs essential for inhibitory signal transduction (5). Its ligands, PD-L1 (CD274, B7-H1) and PD-L2 (CD273, B7-DC), are widely expressed on tumor cells, immune-presenting cells, and stromal cells within peritumoral environment (6, 7). Following ligand engagement, PD-1’s ITSM motif recruits phosphatases SHP-2 (occasionally SHP-1). This recruitment suppresses critical downstream pathways including PI3K/AKT/mTOR and Ras/MAPK cascades, promoting T cell exhaustion, cell-cycle blockade, and apoptotic death (8, 9). Tumors subvert the PD-1 checkpoint—essential for physiological immune tolerance—to avoid detection. Key drivers include: 1) oncogenic pathways (PI3K/AKT, JAK/STAT), 2) inflammatory cues (IFN-γ), and 3) defective ubiquitination (FBXO38-related) promoting stability of the coinhibitory receptor while upregulating its cognate ligand in lymphocytes within the tumor microenvironment, exacerbating immune evasion (5). Thyroid cancer studies confirm blockade therapy restores cytotoxic T cell function and counteracts immunosuppression (10). These agents demonstrate survival extension and enhanced quality-of-life metrics in recurrent/metastatic disease, positioning them as promising clinical interventions. This analysis synthesizes mechanistic principles and therapeutic progress in PD-1/PD-L1 inhibition while proposing strategies to circumvent current therapeutic limitations.

## 2 Method

This narrative review describes the latest advances in PD-1/PD-L1 pathway inhibitors for the treatment of thyroid cancer. The review was conducted following the SANRA (Scale for the Assessment of Narrative Review Articles) guidelines (11). As of May 2025, studies were searched via the PubMed database using the primary keywords “PD-1/PD-L1”, “PD-1/PD-L1 inhibitors”, “thyroid cancer”, “papillary thyroid carcinoma”, “follicular thyroid carcinoma”, and “anaplastic thyroid carcinomas” among others, with no restrictions on study types. A total of 783 publications were initially identified. After removing duplicates and screening through full-texts, abstracts, keywords, and titles, 51 studies meeting the criteria were included.

Reviewers (Li XiZi and Gao SiYuan) independently extracted and synthesized data on PD-1/PD-L1 in thyroid cancer research. Discrepancies were resolved through consultation with a third reviewer (Yu Jiangyi). Data were qualitatively synthesized and presented narratively, supplemented with tables.

## 3 PD-L1 expression in thyroid cancer

Significantly elevated PD-L1 immunopositivity is observed in thyroid cancer versus benign thyroid lesions, particularly in invasive subtypes. A study of surgically excised thyroid specimens revealed significantly elevated cytoplasmic PD-L1 levels in invasive encapsulated follicular variant papillary thyroid carcinomas (IEFVPTC) versus non-invasive follicular thyroid neoplasms with papillary-like nuclear features (NIFTP). This PD-L1 elevation was associated with a 3.16-fold increased risk of invasion, supporting its role as a potential biomarker for aggressive behavior in IEFVPTC subtypes (12).

Significant heterogeneity exists in PD-L1 expression across thyroid carcinoma subtypes. Analysis of 407 specimens by Ahn et al. revealed immunohistochemical positivity rates of 6.1% in PTC, 7.6% in FTC, and 22.2% in ATC. PD-L1 expression was associated with lymph node metastasis, extrathyroidal extension, and the BRAF V600E mutation; however, it was absent in poorly differentiated thyroid carcinoma(PDTC) (6). However, reported PD-L1 positivity rates in thyroid cancer vary widely across studies, ranging from 6.1% to 82.5% (13–16). Chronic lymphocytic thyroiditis (e.g., Hashimoto’s thyroiditis) with eosinophilic immune infiltration may modulate PD-L1 expression in PTC, which complicates interpretation (17). Further research is essential to establish PD-L1’s prognostic relevance in thyroid cancer based on available data. Multiple investigations correlate PD-L1 expression with poor clinical outcomes. Harahap et al. analyzed 26 high-grade and 26 low-grade thyroid cancer samples and found that elevated PD-L1 expression was significantly associated with increased invasiveness and metastasis (18). Another study involving 185 PTC cases demonstrated that PD-L1-positive tumors, particularly those with cytoplasmic localization, exhibited shorter disease-free survival and higher recurrence risk (15). In contrast, Fadia et al.’s analysis of 81 PTC specimens revealed no correlation between PD-L1 levels and either tumor progression extent or high-risk clinical parameters (19), suggesting caution in using PD-L1 as a universal diagnostic marker. These inconsistencies could originate from clinical trial design heterogeneity, antibody clones, detection platforms, cell types analyzed, and scoring criteria. Moreover, autoimmune thyroid diseases may yield false-positive PD-L1 results (20). To enhance diagnostic accuracy, PD-L1 detection methods require standardization, while integrated multi-omics predictive models need development. Specifically, standardization should include:1) membrane-localized staining techniques, 2) consistent scoring protocols (covering staining localization, cell types, and thresholds).

## 4 Regulatory mechanisms of PD-L1 expression

### 4.1 BRAF V600E mutation

**Figure 1:**
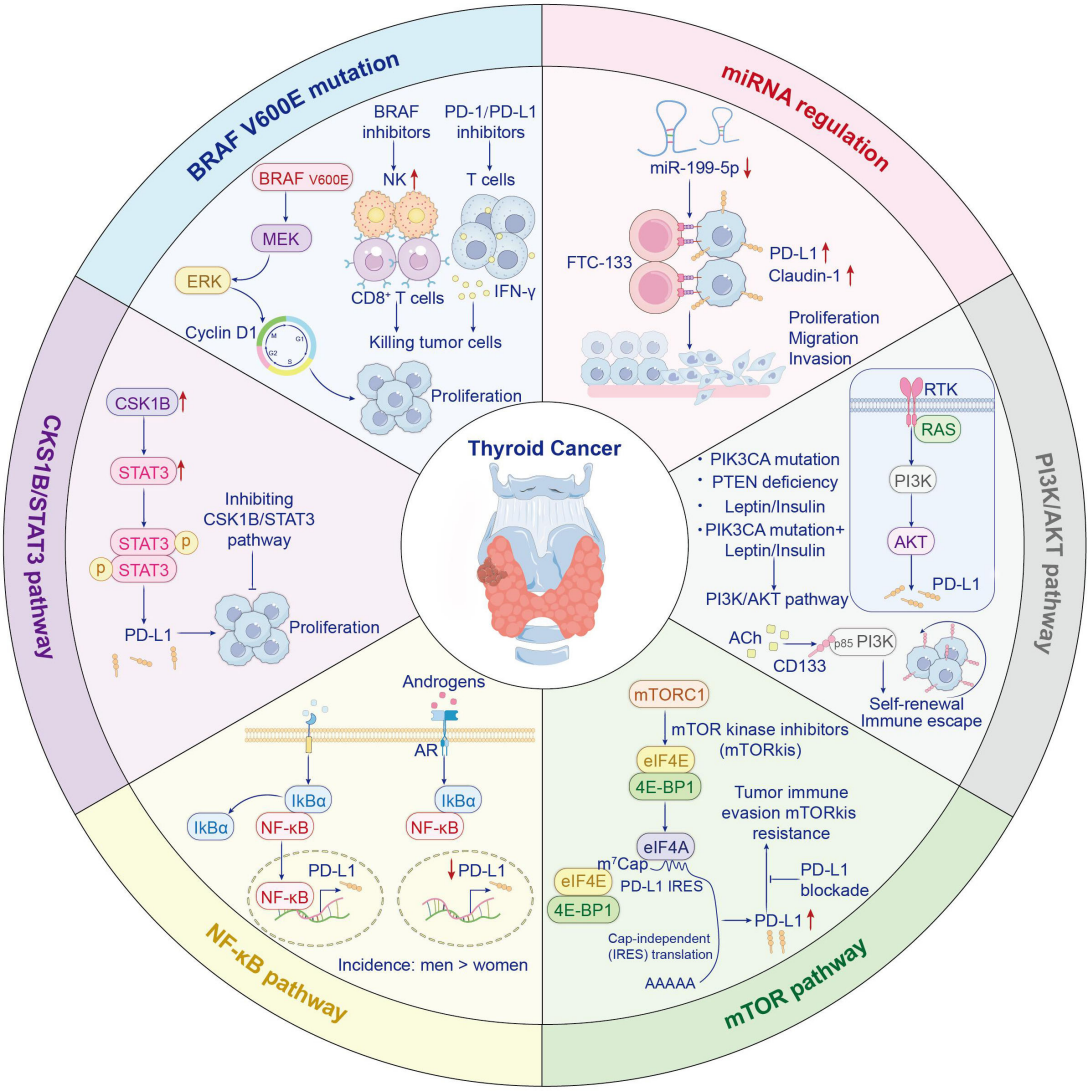
Regulatory mechanisms of PD-L1 expression.

The BRAF V600E mutation drives cell proliferation by activating the MEK/ERK signaling cascade and upregulating cyclin D1, while initiating tumorigenesis in murine models. The BRAF V600E mutation is positively correlated with PD-L1 expression levels in thyroid cancer (21). Byun et al. analyzed ATC patient samples harboring BRAF V600E mutations and, using scRNA-seq, found significantly elevated CD274 and related gene expression scores (22). Additionally, BRAF V600E drives the formation of an immunosuppressive tumor microenvironment (23). In a BRAF V600E-driven ATC mouse model, Gunda V. et al. demonstrated that BRAF inhibitors (including PLX4720) suppress the MAPK pathway, leading to increased natural killer cell infiltration (24). Notably, BRAF-targeted therapy elevates tumor cell PD-L1 expression, exacerbating T cell exhaustion. Concurrent immune checkpoint blockade neutralizes immunosuppression, augmenting antitumor responses. This approach significantly promotes CD8^+^ T cell infiltration and augments functional activity (like granzyme B and IFNγ), ultimately resulting in tumor regression and prolonged survival (25) (**Figure 1**).

### 4.2 MicroRNA regulation

MicroRNAs serve as small non-coding RNA molecules that modulate gene expression and influence immune checkpoint proteins (26). Tumor-suppressive microRNAs contribute to anti-tumor immunity by inhibiting checkpoint proteins. Research has demonstrated the restoration of miR-199a-5p expression has been found to suppress both PD-L1 and Claudin-1, thereby inhibiting thyroid cancer cell proliferation, migration, and invasion (27) (**Figure 1**).

### 4.3 PI3K/AKT pathway

As a master controller of cell growth and survival, the PI3K/AKT axis is frequently dysregulated in cancers. It can be activated by RAS or receptor tyrosine kinases, leading to PIP3 generation and AKT phosphorylation. Downstream, AKT activates mTOR and other effectors. PTEN acts as a tumor suppressor by degrading PIP3; mutations in PIK3CA or PTEN loss lead to sustained pathway activation and tumor progression (28). Research confirms leptin and insulin dose-responsively upregulate PD-L1 via PI3K/AKT signaling in thyroid cancer cell models. This effect is further enhanced when activating PIK3CA mutations, like E545K, are present. Thus, targeting leptin/insulin signaling or the PI3K/AKT pathway may enhance PD-1 blockade efficacy, especially in obese patients or those harboring PIK3CA mutations (29). Moreover, acetylcholine, a neurotransmitter secreted by the tumor neuroenvironment, promotes cancer stem cell self-renewal and immune evasion by activating the CD133–AKT–PI3K axis (30) (**Figure 1**).

### 4.4 MTOR pathway

The mTOR pathway governs cellular proliferation and transcriptional control, with frequent hyperactivation observed in thyroid malignancies. It promotes the nuclear relocation of transcription factors that attach to the PD-L1 promoter (31). The mTOR pathway is frequently hyperactivated in thyroid cancer. Inhibition of this pathway suppresses the growth of MTC and ATC cells in a significant dose-dependent manner (32, 33). Although mTOR inhibitors primarily exert their antitumor effects by suppressing cap-dependent translation, it is hypothesized that they may simultaneously induce immune resistance by specifically upregulating PD-L1 expression through IRES-mediated cap-independent translation (34) (**Figure 1**). Consequently, combining anti-PD-L1 antibodies with mTOR inhibitors may enhance antitumor efficacy and overcome this resistance. A phase II trial evaluated everolimus monotherapy in patients with locally advanced or metastatic thyroid cancer. Limited antitumor activity was observed: only 2 of 31 patients achieved confirmed objective responses, with disease control in 31 cases. The median progression-free survival (mPFS) was 47 weeks across all patients (35). Everolimus demonstrates limited activity in locally advanced or metastatic thyroid cancer, with low response rates. Its reasonable clinical benefit rate and safety profile may warrant further study. The compensatory activation of distinct pro-survival signaling pathways following mTOR inhibition suggests that these adaptive responses may drive tumor progression, highlighting the potential inadequacy of single-agent targeted therapies to control heterogeneous signaling networks within tumor populations. A phase II trial of sorafenib plus temsirolimus for RAI-refractory DTC yielded favorable response rates (36). The above studies demonstrate that combining mTOR inhibitors with PD-1/PD-L1 inhibitors enhances antitumor efficacy.

### 4.5 NF-κB pathway

Thyroid cancer exhibit constitutively active NF-κB signaling, especially PTC and ATC. This activation is induced by receptor stimulation at the cell membrane, triggering NF-κB dimer nuclear translocation and then upregulating pro-tumorigenic genes (37). Research has revealed that the significantly lower incidence of PTC in males compared to females may be associated with androgen signaling modulating the tumor immune microenvironment through suppression of PD-L1 expression (38–40). In androgen-sensitive thyroid cancer cells, dihydrotestosterone (DHT) markedly reduces cell surface PD-L1 levels in both time- and dose-dependent manners. Mechanistically, upon AR activation, upregulated expression of IκBα (an NF-κB inhibitor) blocks nuclear translocation of NF-κB, ultimately suppressing PD-L1 promoter activity and attenuating NF-κB-mediated transcriptional drive of PD-L1 (41) (**Figure 1**). Notably, however, while inhibiting PD-L1, the net effect of androgen signaling is to promote an immunosuppressive tumor microenvironment. This manifests as accelerated CD8^+^ T-cell exhaustion, thereby facilitating tumor growth. Correspondingly, castration has been shown to enhance the anti-tumor efficacy of anti-PD-1 antibodies, indicating that reduced androgen signaling diminishes T-cell exhaustion and sensitizes hosts to more effective immune checkpoint blockade therapy (42).These mechanisms may partially explain the apparent paradox wherein young males exhibit lower thyroid cancer incidence than females yet present with more advanced disease stages at diagnosis. Currently, the role of androgens in thyroid carcinogenesis remains incompletely explored. Inconsistent findings across studies likely stem from variations in experimental conditions, research protocols, and model systems/methodologies.

To address these knowledge gaps, future investigations should employ high-throughput approaches—including genome-wide association studies (GWAS), mRNA sequencing (mRNA-Seq), and ribosome profiling (Ribo-Seq)—to systematically delineate sex-specific regulatory pathways in thyroid cancer. Further integration of spatial omics technologies will help map the sex-dimorphic distribution of immune cells and elucidate androgen-driven mechanisms shaping the gender-specific tumor immune microenvironment.

### 4.6 CKS1B/STAT3 pathway

CKS1B/STAT3 signaling promotes PD-L1-mediated tumor progression in PTC, with coordinated upregulation of CKS1B and PD-L1 observed in cell models at both mRNA and protein tiers. Inhibition of CKS1B impairs STAT3 signaling and suppresses PD-L1 expression, suggesting a novel therapeutic axis for targeting aggressive thyroid cancers (43) (**Figure 1**).

## 5 Anti-PD-1/PD-L1 therapeutics: clinical implementation strategies

### 5.1 Single-agent PD-1/PD-L1 immune checkpoint inhibitors

The study of monotherapy with PD-1/PD-L1 inhibitors in treating thyroid cancer has seen gradual progress, primarily focusing on pembrolizumab and spartalizumab. Pembrolizumab, a fully humanized monoclonal antibody targeting PD-1, can block the interaction between PD-1 and its ligands, thereby exerting antitumor effects (44). A trial first assessed pembrolizumab monotherapy in PD-L1-positive advanced DTC. A nonrandomized phase Ib KEYNOTE-028 trial included 22 patients, among whom some did not receive prior systemic therapy. Results showed an objective response rate (ORR) of 9%, a mPFS of 7 months, and a high incidence of treatment-related adverse events (45) (**Table 1**). In the phase II KEYNOTE-158 trial, the thyroid cancer cohort enrolled 103 patients. Results showed an ORR of 6.8%, a mOS of 34.5 months, and a mPFS of 4.2 months. Treatment-related adverse events occurred in 69.9% of the cohort. Although the ORRs in PD-L1–positive patients were similar across the two studies, differences existed in the study populations. In the KEYNOTE-028 trial, which enrolled exclusively PD-L1–positive patients (including some treatment-naïve individuals), differences in sample size and patient characteristics could have confounded outcome interpretations (46) (**Table 1**). The anti-PD-1 humanized IgG4 antibody Spartalizumab was the first ICI with demonstrated efficacy against ATC. Capdevila et al. enrolled 42 patients with metastatic ATC in a phase II trial. Results showed that the response rate among PD-L1–positive patients (expression ≥1%) was approximately 30%, with good overall tolerability (47) (**Table 1**). Atezolizumab, a PD-L1 inhibitor, prevents PD-L1/PD-1 interaction, disrupting inhibitory signaling in T lymphocytes. In a study involving 11 patients with advanced thyroid cancer (7 with PTC and 4 with FTC), atezolizumab crossed the prespecified interim benefit threshold, although the study was terminated early due to disease progression (48). It inform that PD-1/PD-L1 ICIs demonstrate manageable safety profiles and modest antitumor activity. However, their clinical application may be constrained by low response rates and limited survival benefit. Additionally, small sample sizes and the influence of concomitant treatments may affect the interpretation of these results.

**Table 1:** PD-1/PD-L1 clinical research.

Year	Author	Target	TC Type	N	Intervention	Efficacy	AECommon adverse reactions	Reference
2019	Mehnert JM	PD-1	PTC(N=15),FTC(N=7)	22	Pembrolizumab 10 mg/kg IV q2w for 24 months	ORR:9%,mPFS: 7mo	Diarrhea (32%) Fatigue (18%) Pruritus (14%) Rash (14%) Decreased appetite (9%) Headache(9%) Cough (9%) Pneumonitis (9%) Colitis (5%)	(45)
2023	Oh DY	PD-1	Thyroid Carcinoma(N=103)	103	Pembrolizumab 200 mg IV q3w ×35 cycles	ORR: 6.8%,mDoR: 18.4 mo	Diarrhea (32%) Fatigue (18%) Pruritus (14%) Rash (14%) Decreased appetite (9%) Headache (9%) Cough (9%) Pneumonitis (9%) Colitis (5%)	(46)
2020	Capdevila	PD-1	ATC(N=42)	42	Spartalizumab 400 mg IV q4w	ORR:19%,mPFS:1.7 mo,mOS:5.9 mo	Diarrhea(11.9%) Pruritus(11.9%) Fatigue(7.1%) Pyrexia(7.1%) Anemia(4.8%) Asthenia(4.8%) Myalgia(4.8%) Rash(4.8%)	(47)
2024	Chen JY	BRAF/MEK/VEGF+PD-1	ATC(N=42)	42	Cohort 1:Vemurafenib 960 mg oral BID (Days 1–14 alone; Day 15 onward combined)+Cobimetinib 60 mg oral QD (21/7 schedule) (Days 1–14 alone; Day 15 onward combined)+Atezolizumab 1200 mg IV q3w (Starting Day 15) Cohort 2:Cobimetinib 60 mg oral QD (21/7 schedule)+Atezolizumab 1200 mg IV q3w Cohort 3:Bevacizumab 15 mg/kg IV q3w+Atezolizumab 1200 mg IV q3w Cohort 4:Atezolizumab 1200 mg IV q3w+nab-Paclitaxel per standard IV q3w or Paclitaxel per standard IV q3w	(mOS:19 mo;cohort 1:43mo;cohort 2:8.7 mo;cohort 3:6.21 mo); (PFS:cohort 1:13.9 mo;cohort 2:4.8 mo;cohort 3:1.3 mo)	Colitis (2 case) Papilledema (1 case) Retinopathy (1 case) Pancreatitis (4 cases) LV dysfunction/reduced EF (2 cases) Esophageal perforation (1 case)	(51)
2021	Cabanillas ME	TKI+PD-1	ATC(N=6),PDTC(N=2)	8	BW>80kg,Lenvatinib 24mg QD,Pembrolizumab 200mg q3w BW ≤ 80kg,Lenvatinib 20mg QD,Pembrolizumab 200mg q3w	ATC: ORR:66%;All Patiens:mPFS:17.8 mo	Hypertension(63%) Fatigue(25%) Anorexia(25%) Oral mucositis(25%) Hand–foot syndrome(13%) Diarrhea(13%) Proteinuria(13%)	(52)
2022	Ji D	TKI+PD-1	Radioactive iodine-refractory DTC,DTC not candidates for RAI therapy,	74	Camrelizumab 200 mg IV q3w+Famitinib 20 mg QD	Confirmed ORR: Group 1:33%,Group 2:44%,Group 3:40%,Group 4:63%	Diarrhea (37.0%) Palmar-plantar erythrodysesthesia syndrome (34.2%) Hypertension (31.5%) Fatigue (31.5%)	(53)
2024	Hamidi S	BRAF+MEK+PD-1	BRAFm-ATC patients treated with first-line BRAF-directed therapy,DT Group(n = 23),DTP Group(n = 48),Neoadjuvant Group(n = 23)	71	DT Group: DT therapy only DTP Group: DT + anti-PD-1 ICI Neoadjuvant Group: Neoadjuvant DT + Definitive surgery + Perioperative pembrolizumab	DT:mOS:9.0 mo,ORR:64.5%;DTP: 17 mo,Neoadjuvant: ORR:73.3%;mOS:63.0 mo	Hepatitis(9%) Colitis(6%) Nephritis(4%)	(54)
2019	Wang JR	BRAF+MEK+PD-1	BRAF V600E-mutant ATC(N=3)	3	DT + Surgical Resection + Chemoradiotherapy(Four patients received pembrolizumab)	prolonged DFS	Not Provided	(55)
2024	Sehgal K	PD-1+CTLA-4	Aggressive thyroid carcinoma(N=49,Subtypes Included RAIR-DTC,MTC,ATC)	49	Nivolumab 3 mg/kg IV q2w+Ipilimumab 1 mg/kg IV q6w	ORR:9.4%,mPFS:7.1 mo,mOS:24.6 mo	increased lipase(12%) increased aspartate aminotransferase(6%) increased alanine aminotransferase(8%) increased amylase(48%) increased lipase(6%) hyperglycemia(2%) hypophysitis(2%)	(57)
2025	Fan S	PD-1+CT	PD-L1(+) metastatic ATC(N=1)	1	1st line: paclitaxel liposome +cisplatin x6 cycles followed by paclitaxel 2nd line: gemcitabine+ toripalimab 3rd line: gemcitabine+ S-1 x3cycles followed by S1 4th line: zimberelimab+nab-paclitaxel Zimberelimab resumed as maintenance therapy	long-term remission for 34 months	hypothyroidism	(58)
2021	Yang SR	PD-1+RT	ATC(N=1)	1	Post-radioiodine therapy+Concurrent radiotherapy+Pembrolizumab 2 mg/kg IV q3w	Cervical lymph node regression, no recurrence observed	Grade 1 hepatitis and adrenal insufficiency(after pembrolizumab)	(59)
2023	Xing Y	PD-1+RT	ATC(N=1)	1	RT+Tislelizumab 200 mg IV every 3 weeks	significant tumor shrinkage	No adverse reactions were observed	(60)

### 5.2 Targeted combination immunotherapy

Targeted combination immunotherapy exerts a synergistic effect by directly inhibiting tumor growth while simultaneously activating immune responses. And Targeted agents can induce immunogenic cell death and release neoantigens, thereby enhancing the antitumor efficacy of ICIs (49). Preclinical research has shown that the combination of lenvatinib and pembrolizumab effectively reduces tumor burden and prolongs survival in immunocompetent mouse models of ATC (50). One study enrolled 42 patients with ATC and stratified them according to gene mutation types (BRAF V600E, RAS/NF, and wild-type) for targeted therapy with, respectively, vemurafenib/cobimetinib, cobimetinib, or bevacizumab, each combined with the PD-L1 inhibitor atezolizumab. Studies indicate that combination therapy with a PD-L1 inhibitor significantly extended patients’ mOS compared to historical controls (51) (**Table 1**). Another retrospective study by Cabanillas ME et al. confirmed that targeted-immunotherapy combination regimens were both safe and effective in patients with ATC or PDTC, with some individuals achieving complete and durable remission (52). Additional studies suggest that regimens such as camrelizumab combined with famitinib and surufatinib combined with toripalimab have shown promising antitumor activity (53) (**Table 1**). Dabrafenib plus trametinib is FDA-approved for BRAF V600E-mutant ATC with unresectable/metastatic disease in radiotherapy-ineligible patients. However, drug resistance and disease progression may eventually occur in some patients. Drug resistance remains a significant barrier to combination therapies, and the underlying molecular mechanisms are still not fully elucidated. A retrospective study indicated that combining dabrafenib, trametinib, and pembrolizumab significantly prolonged survival in patients with BRAF-mutant ATC. Although combination therapy increased toxicity, it remained within a manageable range (54) (**Table 1**). Furthermore, a case report described three ATC patients with BRAF V600E mutations who experienced prolonged progression-free survival following treatment with dabrafenib, trametinib, and pembrolizumab (55) (**Table 1**).

### 5.3 Dual immune checkpoint inhibition

While PD-1 monotherapy shows limited efficacy in thyroid cancer, dual immunotherapy demonstrates therapeutic efficacy across solid tumor types. Preclinical models—such as patient-derived organotypic tumor spheroids —have demonstrated that dual blockade of PD-1 and CTLA-4 significantly suppresses the upregulation of chemokines such as CCL19 and CXCL13, resulting in enhanced immune activation (56).

To broaden the population that may benefit from immunotherapy, a recent trial evaluated nivolumab plus ipilimumab for advanced invasive thyroid carcinoma. Although the study did not achieve its predefined primary endpoint and did not support further investigation in DTC patients without biomarker-based selection, a clinical benefit rate of 50% was observed in the ATC subgroup, indicating potential therapeutic promise in this population (57) (**Table 1**).

### 5.4 Immune checkpoint inhibition combined with chemotherapy

Chemotherapy primarily activates antitumor immunity by inducing immunogenic cell death, thereby triggering tumor-specific adaptive immune responses. A case reported a male patient with metastatic ATC who achieved sustained remission after receiving zimberelimab (PD-1 inhibitor) in combination with albumin-bound paclitaxel (nab-paclitaxel) for 34 months (58) (**Table 1**). Given the current scarcity of clinical data on this combination regimen—with evidence largely confined to case reports—rigorous evaluation of its therapeutic efficacy and long-term safety necessitates well-designed prospective trials.

### 5.5 Immune checkpoint inhibition combined with radiotherapy

Radiotherapy induces immunogenic tumor cell death, thereby converting the tumor microenvironment into an immunostimulatory state. Yang et al. reported a patient with BRAF-negative ATC exhibited disease progression following radiotherapy. However, upon following pembrolizumab (PD-1 blockade) administration, the patient attained sustained clinical benefit for >2 years with manageable toxicity. Notably, the largest liver metastasis significantly regressed, and overall disease burden was markedly reduced (59) (**Table 1**). Xing et al. treated an ATC patient with a combination of radiotherapy and tislelizumab, resulting in substantial tumor shrinkage. Regarding combination therapies of immune checkpoint inhibitors with radiotherapy or chemotherapy, most current evidence stems from case reports with a low level of evidence. Future studies should conduct large-scale, multicenter clinical trials with long-term follow-up to determine the efficacy and safety profiles of maintenance regimens (60) (**Table 1**).

### 5.6 Epigenetic intervention combined with immunotherapy

Epigenetic abnormalities, such as hyperactivation of histone deacetylases (HDACs), contribute to the progression and immune evasion of thyroid cancer. HDAC inhibitors can regulate tumor cell cycles and reshape the immune microenvironment by restoring histone acetylation levels (61–63). A novel ATC cell line, PF49, was established from the pleural effusion of a patient harboring BRAF and TERT mutations, revealing the potential of epigenetic agents to sensitize tumors to immunotherapy studies indicate that histone deacetylase inhibitors, including vorinostat (SAHA) and valproic acid, promote PD-L1 upregulation in PF49 cells. This effect is observed when these inhibitors are co-administered with cisplatin and paclitaxel chemotherapy, PD-L1 expression is further enhanced. These findings suggest that epigenetic interventions, such as HDAC inhibition, may enhance the efficacy of PD-1 on tumor cells and thus provide additional immunotherapeutic targets (64)(**Figure 1**).

### 5.7 Exploration of therapeutic strategies

Oncolytic viruses (OVs) constitute a novel cancer immunotherapy capable of selectively infecting and lysing tumor cells with minimal damage to normal tissues (65, 66). In response to the therapeutic challenge of BRAF-mutated ATC, a recent study proposed a three-drug combination regimen (oHSV/BRAFi/anti-CTLA-4). In this regimen, BRAF inhibitors reduce tumor burden. However, the oncolytic herpes simplex virus (oHSV) uses the release of antigens to activate cytotoxic lymphocyte. Meanwhile, matching antibody inhibition reverses the compensatory overexpression of Immune Checkpoint Molecules. Mouse model data showed that this combination prolonged survival, and its efficacy was dependent on T cell and NK cell activity (67). Despite its transformative impact on hematological malignancies, chimeric antigen receptor T-cell (CAR-T) therapy has achieved only limited success in solid tumors, primarily because of the complex tumor microenvironment and surface antigen heterogeneity (68, 69). Identified CAR-T targets include intercellular adhesion molecule-1 (ICAM-1), GDNF family receptor alpha-4 (GFRα4), and the thyroid-stimulating hormone receptor (70, 71). Gray et al. demonstrated that ICAM-1-positive tumor cells could be effectively eliminated by targeting IFNγ-induced ICAM-1 and PD-L1 via a complementary mechanism, thereby alleviating immunosuppression (72). In experimental mouse models, the combination therapy also resulted in rapid tumor shrinkage and prolonged survival, demonstrating its potential to suppress the progression of advanced thyroid cancer. Natural compounds exhibit anticancer potential by targeting the PD-1/PD-L1 immune checkpoint pathway. Using *in vitro* models, silybin was found to inhibit AKT phosphorylation through FN1 downregulation, consequently blocking downstream survival and invasion pathways (e.g., mTOR/GSK-3β) (73). Silybin also significantly decreased mesenchymal. Curcumin reduces PD-L1 expression in ATC cells through inhibition of the AKT/mTORC1/STAT3 signaling pathway; concurrently, the immunosuppressive tumor microenvironment is reversed. Simultaneously, CD8+ T cell function is enhanced (e.g., increased IFN-γ secretion) and tumor infiltration is promoted, resulting in augmented anti-tumor immunity. When combined with anti-PD-1 therapy, a synergistic enhancement of CD8+ T cell activity is observed, and antitumor effects are exerted by curcumin (74).

## 6 Summary

The PD-1/PD-L1 pathway has achieved significant progress in thyroid cancer. However, while PD-1/PD-L1 inhibitor monotherapy demonstrates limited efficacy, combined approaches—such as integrating ICIs with BRAF inhibitors or radiotherapy—have significantly improved therapeutic outcomes. Several clinical trials have even reported unprecedented survival benefits. Moreover, emerging therapies, including oncolytic viruses, epigenetic interventions, and the synergistic use of natural compounds, offer promising new avenues to overcome immune resistance. To combat resistance and improve safety, further studies should explore optimized drug combinations, dosage regimens, and timing strategies. Preclinical evidence indicates synergistic efficacy between immune checkpoint blockade and CAR-T cell immunotherapy, epigenetic regulators (e.g., HDAC inhibitors), and androgen receptor-targeted therapies.

In summary, while PD-1/PD-L1 inhibitors herald a promising frontier, their broad clinical application still requires overcoming numerous mechanistic, technical, and translational hurdles. Through sustained efforts, we hope to achieve durable disease control for patients with thyroid malignancies.
